# Pivot Teeth

**Published:** 1869-12

**Authors:** 


### ARTICLE IX.

### *Pivot Teeth.*

Dr. J. Hardman in a communication to the *Missouri Dental Journal*, says: I notice in the August number of the *American Journal of Dental Science*, page 365, a communication from the pen of J. B. Bean, M.D., upon "Pivot Teeth." It gives me much pleasure to see that this subject is being once again brought before the profession with a prospect of a fair degree of notice.

The time has been when a pivot tooth and the ability to well plant one received quite a share of honorable credit. But for the last ten or fifteen years a great neglect of this variety of work has prevailed. And indeed, we can frequently meet dentists who say they never use pivot teeth, or plant artificial crowns upon the natural roots.

Several causes have contributed to this. Amongst the most prominent is that of cheap dental plates, introduced by the use of rubber. Another is the use of adhesive gold in building out large portion or entire crowns of front teeth. The first of these causes, good and efficient operators will not be influenced by. But the second will require a little more persuasion to obtain an acknowledgement from many, that a porcelain tooth, planted with pivot, ought to be preferred to a gold crown in the front part of the mouth. Of course, no rule can be brought to bear in determining the true relative value of the two modes, as much of the objection rests with the damage the yellow glaring metal has upon the physiogomy of the patient; and which is very much modified in effect by the amount the person exposes the teeth. But all must admit at once, in beholding one of these grinning subjects with a large yellow advertiser in his front teeth. the immense injury done to his natural expression. He strikes you at once as being very proud of the show of jewelry (whether worn in proper place or not) or being rather easily persuaded to be a walking advertiser for his operator.

No one will be likely to deny that a front root sufficiently healthy to bear filling from the apex (with mallet pressure) will, if properly done, equally sustain a pivot tooth. And how immeasurably more preferabe would the porcelain tooth be than the mass of gold.

The third cause, that it is difficult to obtain good pivot teeth (though true) ought not to bear weight in this matter, as the want will most assuredly be supplied by our manufacturers so soon as evidence of their want is made known.

I have tried quite a variety of modes for planting these teeth. However, I have never placed a gold tube in by filling around with gold foil, nor have I fitted a gold pivot to permit daily removal, by the patient, as recommended by Dr. Bean. At present, I think I see no especial advantage in these featnres. The filling around the tube to secure it must necessarily reduce the strength of the root just where it is the most liable of being split in hard biting. And. if the tooth may be removed *ad libitum* by the patient, it is almost absolutely certain that from negligence or otherwise the tooth will be lost, or may be swallowed with the food.

After trying various plans for the use of metal tubes and pivots, I have finally adopted the non-metallic mode and abandoned all other, except where the root is too much out of range, or where much decay has reduced materially the dentinal strength.

My favorite mode after the road is prepared with the excising forceps, file and drill, is to proceed at once to fit a crown mounted upon a temporary pivot made of pine wood.

If a nerve is to be removed, cover it with a little wax, and congeal with atomizing spray and extirpate to the apex. Wipe out and apply a small dossil of cotton wool saturated with creosote with a blunt broach up out of the way of the drill. Then drill first with a conical, and next with a square point, to little more than two lines in depth. Then select a tooth of proper size, shade and form. Also, with correct pivot hole to correspond with the root. This latter is best done by placing a piece of pine or match stick in as a trial pivot. Now, if all is favorable so far, grind the base to fit truly upon the base of the root. To do this, it is a good plan to file with the same convex surface used to file the base of the root, a concave notch in a piece of wood, and by a little experience a good fit is secured by repeated trials to this surface. Then again try on root with pine trial pivot, and see that position, length, &c., are all right. Now cleanse out the canal, which has most likely ceased bleeding from the apex, and place again cotton and creosote in the apex of the root, and plant the tooth upon a pine pivot.

This pivot is prepared by drawing a good piece of white pine through the graded holes of a draw plate until sufficiently condensed. The patient is dismissed for a fortnight or more, with the injunction that if pain or swelling ensue, to return at once.

If upon the return all is going on aright, remove the tooth, clean the canal, and place a dossil of cotton or bibulous paper with creosote well packed into the apex, whether the nerve has recently been removed, or has long since been dead. And over this pack oxychloride zinc, and when sufficiently hard, cleanse out the pivot hole to the proper depth, and replant the tooth with permanent hickory pivot.

To insure success, and the best possible results, every part must be properly adjusted, and the root must be in a good state of health, and the patient enjoying a good constitutionality.

A single pivot tooth, aided by natural teeth upon either side, of course does best. However, I have a few patients who have four to six pivot teeth placed continuously on the front superior roots which have been in service over seven years, and but one or two of the pivots have required renewing.

I have, also, in a few cases engrafted successfully with wood pivot upon the root of the front bicuspid. This root when cylindrical and favorably situated, will bear pivoting. The porcelain canine tooth will be more readily ground in shape for this purpose.

I insist that the profession pay more regard to this variety of artificial teeth. They are the most useful, natural and beautiful.

